# The Treatment of Graves's Disease

**Published:** 1906-01-06

**Authors:** 


### THE TREATMENT OF GRAVES'S DISEASE.

Several interesting contributions have recently been made to the literature of Graves's Disease. In a lecture by Dr. Hector Mackenzie [1] it is recognised that drugs hold a secondary place in the treatment of Graves's disease. On the other hand, rest of mind and body is of prime importance, and so long as the patient is losing flesh bodily rest should be absolute. Good food, cheerful surroundings, and a plentiful supply of fresh air are also of great importance. In reference to the last-mentioned the open-air treatment may be profitably employed, and this is the more easy, as sufferers from Graves's disease are very tolerant of cold, and always feel better in cool weather. In the dietary scheme fat ought to occupy a prominent place, and should there be loss of weight milk should be given freely in addition to the ordinary diet. Massage, warm baths, and saline or brine baths are other useful agencies. Change of air and climate is often of value for patients who are not confined to bed, but no absolute rule can be defined in this matter; one case will do well at the seaside while for another the seaside proves too exciting, and so on with regard to inland and mountainous districts. Care is of far more importance than climate, and peaceful quiet and rest without noise and excitement are essentials. Sea voyages as a rule are prejudicial, except when the patient is well on the road to recovery.

Of drugs potassium bromide is particularly valuable in nervous and emotional patients; it should be given in doses of 20 to 40 grains at bedtime. When the goitre is large or is increasing in size iodine preparations are indicated. Syrup of hydriodic acid in drachm doses thrice daily is better borne than potassium iodide. Iodine tincture or liniment may be applied externally, or for the same purpose 20 minims of iodipin (20 per cent.) may be rubbed into the skin over the enlarged gland every night. Loss of flesh or an increase of the cardiac or nervous symptoms is a signal for the cessation of treatment by iodine. Belladonna is particularly useful in cases where cardiac symptoms predominate; 10 minims of the tincture may be given thrice daily. The administration of the drug should not be continuous but should be suspended from time to time. Some authorities advise digitalis or strophanthus, but whatever value these remedies may have in other directions they seldom or never diminish the tachycardia. Sodium phosphate and calcium chloride are other drugs which sometimes seem to do good. Treatment with thyroid gland usually makes the patient worse, but in certain old standing cases where the symptoms seem to be due rather to deficient than to excessive thyroid secretion, it is usually successful. Thus cases with solid œdema are usually much benefited. Thymus, suprarenal, and spleen extracts have all, in Dr. Mackenzie's experience, proved useless. He has formed the same conclusion in reference to the serum introduced by Professor Möbius. Such local measures as cold compresses or Leiter's tubes over the thyroid, glycerine of belladonna or of atropine over the præcordium are sometimes of value. Electrical treatment is disappointing, and any benefit which follows its adoption is probably due to the effect produced on the patient's mind. In regard to thyroidectomy Dr. Mackenzie's conclusion is that the risk is too great to justify the adoption of the operation save in exceptional circumstances. This last conclusion is combated by Dr. William Hardman,[2] who contends that Kocher's statistics show cure in 76 per cent. of the cases, and improvement in 14 per cent. Dr. Hardman also draws attention to the method of treatment by intraglandular injections of iodoform in ether. He quotes Abadie's and Collon's figures on the results of this method in twenty-four cases: cured 12, notably and permanently improved 9, temporarily improved 3. There were no fatalities. Dr. Hardman's conclusion is therefore opposed to medical treatment and in favour of either thyroidectomy or iodoform injection, with a leaning towards the latter. Mr. William Prowse [3] urges attention to the value of the ointment of red iodide of mercury as an external application in cases both of simple goitre and of Graves's disease. The ointment, having a strength of 5 grains to the ounce, should be gently rubbed in night and morning until the skin begins to peel, when some emollient application should be used. As soon as healing is secured the iodide ointment should again be applied. With this remedy Mr. Prowse reports that he has "never failed to get, sooner or later, a good result." In two cases of exophthalmic goitre the method just described, together with the administration of two minims of Fowler's solution thrice daily, proved quite successful in the course of three months. In his recent Bradshaw lecture Dr. George R. Murray [4] deals with the subject now under review and concludes that with the means now at our disposal it is possible not only to relieve special symptoms but also favourably to modify the course of the malady. After dealing with the importance of general hygiene and of the combination of rest with open-air influences he proceeds to speak of more specialised methods. One of the most valuable of these is the systematic and persevering application of a Faradic current. The most satisfactory procedure is that recommended by Sir Victor Horsley. Two flexible electrodes, each about four by two inches, and covered by flannel or wash leather, are moistened with warm salt solution and applied the one over the goitre and the other at the back of the neck, and fixed in position by straps and buckles. They are then connected with the secondary circuit of a dry-cell Faradic battery provided with a water rheostat, so that the strength of the current may be regulated. The current should be sufficiently strong to produce a prickling sensation in the skin, and should be applied for an hour twice in the day; in some cases the application may be extended over three or even four hours in the day. Dr. Murray records steady improvement and practical recovery in several cases in which this treatment has been systematically pursued. The *x*-rays have been tried in a number of cases, and the results suggest the advisability of further trials. In some cases there can be no doubt that medicinal agents are of decided value. Arsenic in small doses—four or five minims of Fowler's solution—three times a day for six to twelve months, stopping the remedy for a week in each month, is a useful routine prescription. When the pulse rate exceeds 110, 10 or 15 minims of tincture of convallaria should be added to each dose. With much nervousness potassium bromide, 10 grains, should also be given for a few weeks at a time. Belladonna is useful in some cases, and particularly where perspiration is excessive, but is not suitable for prolonged administration. A weak ointment of red iodide of mercury sometimes acts well. The persistent vomiting which is sometimes such a formidable symptom may be met by morphine given hypodermically or in a suppository, and extreme tachycardia may yield to an ice-bag placed on the præcordium. Dr. Murray concluded his lecture with an elaborate statement of the various attempts that have been made to prepare a serum for the treatment of exophthalmic goitre and of the clinical results which have been recorded. In some instances very striking successes have been claimed, but the conclusion must be that "as yet no serum or other animal product can be considered to give better results than the older methods of treatment."

[1] Brit. Med. Jour., Oct. 28, 1905. [2] Ibid., Nov. 4, 1905. [3] Ibid. [4] Ibid., Nov. 11, 1905.

